# Sex and environment shape cochlear sensitivity in human populations worldwide

**DOI:** 10.1038/s41598-025-92763-6

**Published:** 2025-03-26

**Authors:** Patricia Balaresque, Sébastien Delmotte, Franklin Delehelle, Andreia Moreira, Nancy Saenz-Oyhéréguy, Myriam Croze, Tatyana Hegay, Tamara Aripova, Sylvie Le Bomin, Philippe Mennecier, Didier Descouens, Sylvain Cussat-Blanc, Hervé Luga, Angel Guevara, Maria Eugenia D’Amato, Turi King, Catherine Mollereau, Evelyne Heyer

**Affiliations:** 1 Centre de Recherche sur la Biodiversité et l’Environnement (CRBE) UMR5300, CNRS, Université de Toulouse, IRD, Université Paul Sabatier, 118 Route de Narbonne, Bâtiment 4R1, 31062 Toulouse, France; 2 MAD-Environnement, Nailloux, France; 3 Institut de Recherche en Informatique de Toulouse (IRIT) UMR5505, CNRS, Université de Toulouse, Toulouse, France; 4 Laboratorio de ADN del Servicio Nacional de Medicina Legal y Ciencias Forenses, Quito, Ecuador; 5 Forensic DNA Lab, Department of Biotechnology, Faculty of Natural Sciences, University of the Western Cape, Bellville, South Africa; 6 Institute of Immunology and Human Genomics, Academy of Sciences, Tashkent, Uzbekistan; 7 Eco-Anthropologie (EA) UMR7206, Muséum National d’Histoire Naturelle, CNRS, Université de Paris, Musée de L’Homme, Paris, France; 8 Clinique Pasteur, Toulouse, France; 9 Milner Centre for Evolution, University of Bath, Bath, UK; 10 Centre de Recherche sur la Cognition Animale (CRCA-CBI) UMR5169, CNRS, Université de Toulouse, Toulouse, France; 11 Institut de Biologie de l’École Normale Supérieure (IBENS) UMR8197, CNRS, École Normale Supérieure, Paris, France; 12 The International ImMunoGeneTics Information System (IMGT), Institut de Génétique Humaine (IGH) UMR9002, CNRS, Univ. Montpellier (UM), Montpellier, France; 13 Sorbonne Université, 21, Rue de L’école de Médecine, Paris, France; 14 Institut Universitaire de France, Paris, France; 15 Université Jean-Jaurès, Toulouse, France; 16 Instituto de Biomedicina, Universidad Central del Ecuador, Carrera de Medicina, Quito, Ecuador; 17 Department of genetics, University of Leicester, Leicester, UK

**Keywords:** Transient-Evoked Oto-Acoustic Emission (TEOAE), Cochlear sensitivity, Human populations, Variability, Environment, Sex, Auditory processes, Biological anthropology, Evolutionary ecology

## Abstract

Hearing remains an underexplored aspect of human evolution. While the growing prevalence of hearing issues worldwide highlights the need to investigate factors beyond age, ototoxic substances, and recreational noise— factors affecting only a subset of the population —the role of environmental influences remains relatively unaddressed. In contrast, hearing and vocalizations have been extensively studied in many vertebrates through the Acoustic Adaptation Hypothesis, which suggests that acoustic communication adapts to the structure of the immediate environment. To explore how the environment shapes the ear’s ability to process sound, studying the cochlea is essential since it is responsible for capturing, amplifying, and converting sound waves into electrical signals. Cochlear sensitivity can be measured using Transient-Evoked Otoacoustic Emissions (TEOAE), which assess the cochlea’s ability to produce and transmit an acoustic response after sound stimulation. By analyzing TEOAE profiles, we gain valuable insights into how the cochlea responds to external auditory stimuli. We evaluated the influence of both endogenous (age, sex, ear side) and exogenous factors (ethnicity, environment, language) on cochlear sensitivity by collecting TEOAE data from 448 healthy individuals across 13 global populations in Ecuador, England, Gabon, South Africa, and Uzbekistan, living in diverse environments. For each individual, we derived six acoustic metrics from these TEOAE profiles to characterize the amplitude and frequency spectrum of cochlear sensitivity. Our results show that amplitude is primarily influenced by sex (up to 2 dB) and environment (up to 3.6 dB), followed by age and ear side. The frequency spectrum is determined exclusively by exogenous factors, with environment— particularly altitude, and urban versus rural settings —being the most significant. These findings challenge existing assumptions and highlight the need to consider both biological and environmental factors when studying auditory processes.

## Introduction

Hearing remains an underexplored aspect of human evolution. While the growing prevalence of hearing issues^1^ worldwide highlights the need to investigate factors beyond age^2–4^, noise exposure^5,6^ and ototoxic substances^7,8^ — factors that affect only a subset of the population —the role of environmental influences remains relatively unaddressed. In contrast, hearing and vocalizations have been extensively studied in many vertebrates through the Acoustic Adaptation Hypothesis (AAH)^9^, which posits that habitat characteristics, such as vegetation density, drive the evolution of auditory adaptations, enabling animals to optimise communication in specific environments^9,10^. While primarily developed through studies of non-human primates^11–17^, indirect evidence from human populations, such as the influence of temperature on language sonority^18^, suggests that environmental variables may also influence human acoustic communication. However, the mixed results reported in the literature^10,19^ on vertebrates emphasize the need for integrative studies that jointly consider biological traits, ecological contexts^10,20,21^, and environmental pressures.

The cochlea plays a central role in the auditory process, capturing, amplifying, and converting sound waves into neural signals, thus acting as a critical interface between humans and their environment. The term “cochlear sensitivity” is adopted here to reflect what is measured by the Transient-Evoked Otoacoustic Emissions (TEOAE) techniques – that is, the cochlea ‘s ability to produce and transmit an acoustic response after being sound-stimulated. Cochlear sensitivity varies across individuals due to biological factors such as age, ear side, and sex. For example, sensitivity declines with age, the right ear is typically more sensitive than the left^22–24^, and women generally exhibit higher sensitivity than men particularly above 2000 Hz^24–26^, although this sex difference is not always consistently observed^27,28^. Additionally, ethnicity has been linked to differences in auditory sensitivity^4,29^, suggesting evolutionary adaptation to local environments^30^. Despite this, the extent to which both endogenous (e.g., sex, age, ear side) and exogenous (e.g., ethnicity, environment, culture) factors jointly shape cochlear sensitivity in humans remains underexplored.

This study aims to analyze cochlear sensitivity across global human populations and evaluate the relative impact of both endogenous and exogenous factors on its variability^31^. Using TEOAE measures, we analysed 448 profiles from healthy individuals across 13 populations in Ecuador, England, Gabon, South Africa, and Uzbekistan. These populations were selected to capture a wide range of ecological and cultural contexts, including underrepresented rural and non-European groups. By examining variations in cochlear sensitivity and the frequency range, we aim to elucidate how biological and environmental factors contribute to shaping auditory adaptations in humans.

## Materials and methods

### Terminology used

Hearing is a complex process that involves multiple levels of the body, ranging from the peripheral system to the central nervous system. Consequently, the terms ‘auditory sensitivity’ and ‘hearing sensitivity’ encompass a variety of concepts and measures, which are interpreted differently across scientific disciplines. In this article, we use the term ‘cochlear sensitivity’ specifically to refer to the measures of Transient Evoked Otoacoustic Emissions (TEOAEs), which reflect the activity of the outer hair cells of the cochlea.

### Population sampling and informed consents

A total of 448 healthy individuals from 13 populations living in different environments in five countries—Ecuador, England, Gabon, South Africa and Uzbekistan—were sampled (Table 1). All participants signed an informed consent. For each individual, biological data—including sex and age were collected. Each participant filled a questionnaire including different sections. The questionnaires were subsequently anonymized and stored according to the relevant guidelines of the French ethical committee (Ile de France VIII, number 2023-A00474-41 ANSM declaration ID RCB 2023-A00474-41). In the present study, we used the following sections: genealogical ancestries, general health status (and potentially associated intake medications: antibiotic, chemotherapy drug, thyroid treatment), hearing health (familial history of hearing loss, cochlear implant, tympanic damage), general health (cancer, thyroid diseases, ENT disorders, etc.) and listening habits. We haven’t included in our sample, people reporting family history of hearing loss, having cochlear implant, showing eardrum damage, and recent otitis media. Similarly, individuals taking ototoxic medications, antibiotics, or any treatment possibly interfering with TEOAE were not considered. .

**Table 1:** Populations and sampling information.

Code pop	Population	Country	Mother language	Family language class	Environmental elements	N	Nf	Nm
AND	Teligote	Ecuador	Quechua, Spanish	Amerindian & Indo-European	Rural-High altitude	36	19	17
BUK	Bukhara	Uzbekistan	Uzbek, Tajik	Altai & Indo-European	Urban-steppes	46	23	23
CHA	Chateauville	Gabon	Fang	Niger-Congo	Rural-forest	24	13	11
COL	Colta	Ecuador	Quechua, Spanish	Amerindian & Indo-European	Rural-High altitude	47	23	24
CPT	Cape Town	South Africa	English	Indo-European	Urban-plains	45	25	20
DOU	Doumassi	Gabon	Baka	Niger-Congo	Rural-forest	27	18	9
ESG	Essang	Gabon	Baka	Niger-Congo	Rural-forest	29	14	15
ESS	Essicot	Gabon	Fang	Niger-Congo	Rural-forest	13	7	6
LEI	Leicester	England	English	Indo-European	Urban-plains	42	23	19
MEB	Meubeum	Gabon	Fang	Niger-Congo	Rural-forest	19	12	7
NUK	Nukus	Uzbekistan	Karakalpak	Altai	Urban/Rural-Steppes	53	29	24
QUI	Quito	Ecuador	Spanish	Indo-European	Urban-altitude	50	25	25
XHO	Cape Town Xhosa	South Africa	Xhosa	Niger-Congo	Urban-plains	17	11	6
All populations						448	242	206

### Ethical approvals

Research procedures were approved by the relevant ethics committees in Uzbekistan (University of Tashkent, N°4/1255–2665), England (University of Leicester Ethics Committee; n°10,464 Cancer Research Center), South Africa (Biomedical Science Research Ethics Committee of the University of the Western Cape, BM18/5/4), Ecuador (Ethical committee for Human research, Central University of Ecuador [CEISH-UCE], No. 356-CEISH-UCE-2022 and N° 021-CEISH-UCE-2022) and Gabon (University Omar Bongo of Libreville SJ N°1165/16, framework agreements between the University Omar Bongo of Libreville and the National Museum of Natural History, Paris, France). Research was performed in accordance with respective relevant guidelines.

### TEOAE’s measure choice and principle

Oto-acoustic emissions, such as Transient-evoked Oto-Acoustic Emissions (TEOAE), are a non-invasive measure that reflect the biological capacity of the inner ear to receive and amplify an acoustic signal from the external environment, which is then transmitted as an electrical signal to the brain. The TEOAE measure involves presenting click-stimuli via an emitter and recording the response signal generated by the outer hair cells in the cochlea, which vibrate in response to the stimulus. The greater the vibration of the stereocilia in the outer hair cells, the higher the returned signal. A schematic of the apparatus is provided in the supplementary material 1 (supp. Figure 1).

All participants underwent a visual otoscopic examination of the ear canal and tympanic membrane to exclude external or middle ear pathology. To minimise external noise during data collection, TEOAE were acquired in the quietest location at each site, and participants wore BOSE® headphones in passive mode to attenuate external noise. Three TEOAE measures were recorded from each ear using an Echoport ILO 288OAE device (Otodynamics Ltd, Hatfield, UK, Amplifon) and the software ILO V6.41 (EZ Screen ILOv6) with the quick-screen default setting. A custom ear probe equipped with a miniature speaker and a sensitive microphone was used to deliver auditory stimuli and record cochlear responses. The probe was always calibrated before examinations with the Otodynamics probe test cavity. The ILO V6.41 system uses a non-linear wide-band click stimulation mode, and we set the target stimulation at a mean intensity of 84 dB p.e.SPL. Since the response emission contains the same frequencies as the stimuli, distinguishing the response emission can be challenging: multiple repeated stimuli are required and averaged to differentiate the response emission from the initial stimulus. For this purpose, the responses were stored in two buffers (S1, S2), and by averaging procedures, the OAE response is separated from noise. High correlation between S1 and S2 indicated a consistent otoacoustic response, reducing the likelihood of noise contamination. Correlation thresholds were set at > 70% to ensure data reliability. We used the raw data provided by the software (data file extension: *.dta) and analysed them using MATLAB. The signal-to-noise ratio (SNR) was calculated by comparing the amplitude of the TEOAE response within the target frequency band ((S1 + S2)/2) to the baseline noise level ((S1—S2)/2). An SNR threshold of 3 dB across the whole profile was established as the minimum for TEOAE detection, distinguishing true emissions from background noise, and indicating functional cochlear outer hair cell activity. A total of 118 frequency points from 546.88 to 5117.19 Hz were selected for further analyses. A preprocessing step excluded aberrant records, resulting in the removal of two individuals, leaving a total of 448 individuals, out of the 450 initially sampled, to be included in the following analyses. Before proceeding with our analyses, we confirmed that stimulation and mean amplitude were not correlated (correlation = 0.0145, t = 0.77, p = 0.44).

### Data processing

**Fig. 1:**
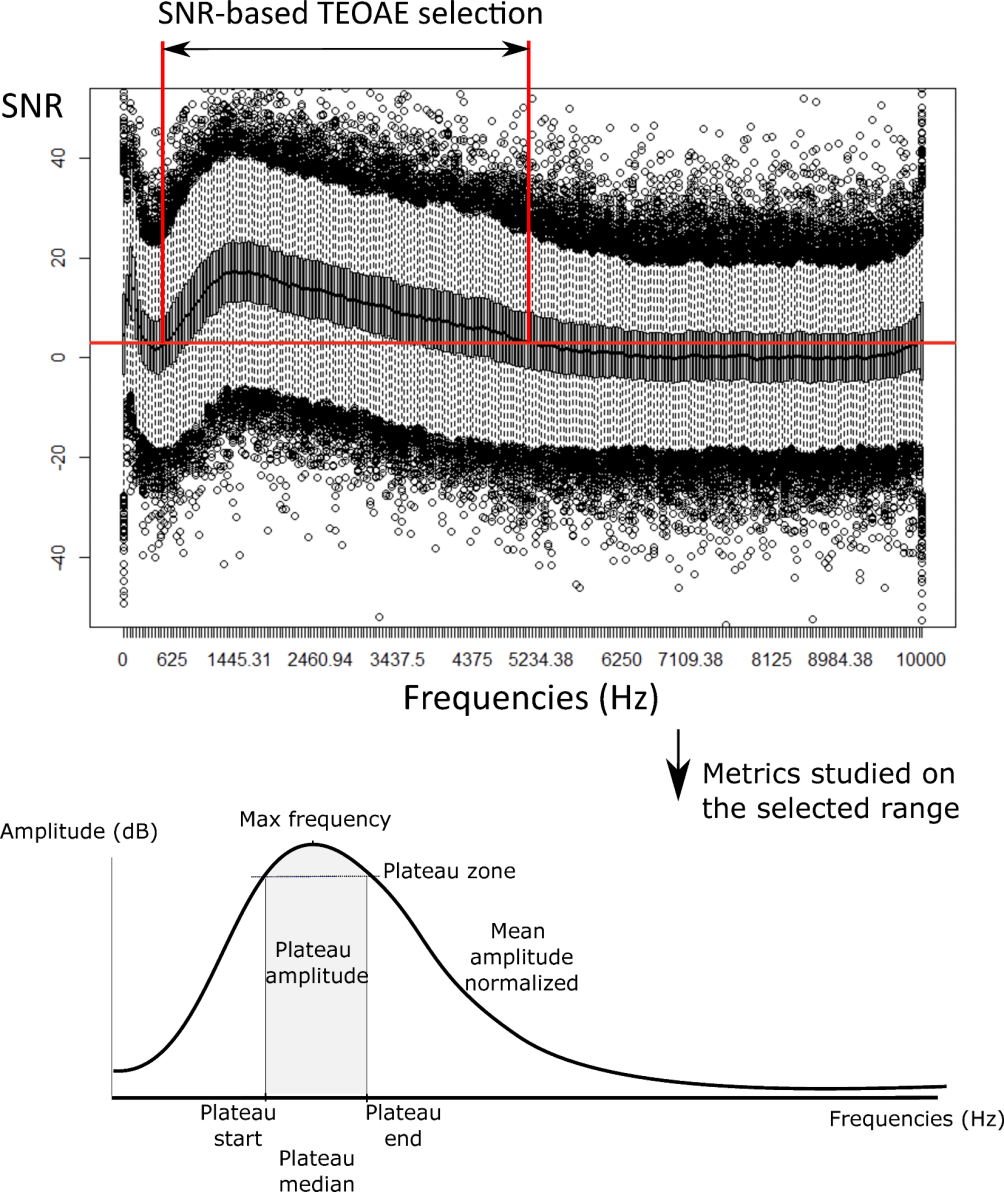
All TEOAE profiles and implementation of a median SNR values > 3 to select the range of frequencies for further analyses; b) Schematic representation of the TEOAE derived metrics analysed in the paper.

Several metrics were extracted from different sections of the TEOAE profiles (Fig. 1), including mean amplitude (in dB), plateau mean amplitude (defined as the mean amplitude calculated over frequencies where amplitudes exceed 90% of the maximum amplitude, in dB), plateau start, median, and end frequencies (in Hz), and maximum frequency (in Hz).

### Statistical analyses

We tested the impact of two categories of factors: endogenous and exogenous. Endogenous factors included biological variables directly related to each individual analysed: sex (Male, Female), age (4 age-classes: [18-25], [25-35], [35-45], [45-55]), and ear side (Left, Right) (categorisation in supplementary material 1, supp. table 1). Exogenous factors encompassed non-biological variables potentially influencing variations in cochlear sensitivity. Populations (equivalent to ethnic group) were considered as an exogenous factor and were later reclassified into environment type and further into language families, all serving as alternative levels for grouping individuals. In this study, we referred to these three levels of grouping as the population-based model (a), the environment-based model (b), and the language-based model (c).

The environment was characterised using six parameters describing the ecological landscape associated with each population, which affect sound propagation (Table 2). These parameters, estimated either directly or indirectly using the Global Cover website^25^, included altitude (in meters), level of artificialisation (low, medium, high), forest coverage (absent, low, medium, high), vegetation diversity (low, medium, high), bare soil coverage (absent, low, medium, high), and population density (hab/km^2^). These were combined into a global index using Hill and Smith PCA^32^, and a classification tree (Hierarchical Ascendant Classification using Ward’s distance) was built using the first three PCA axes. We focused on a three-class typology corresponding to the most prevalent environments in our dataset: C1 – urban zone, C2 – rural high altitude zone, and C3 – rural natural-forest zone. This newly defined variable, labelled ‘Environment’, was used in subsequent statistical analyses (Table 2). Language was also considered as an alternative exogenous factor for grouping individuals: we categorised individuals based on their language family (native language), resulting in four main language families: Amerindian, Niger-Congo, Indo-European, and Turko-Mongol (Table 1).

**Table 2:** The 6-parameters defining 3 classes of environment typology used in Hill and Smith PCA analyses.

Code pop	Altitude	Artificial	Forest	Div Veg	Bare	Density	Environment class considered
AND	2880	Low	Medium	High	Abs	Low	C2
BUK	225	Medium	Abs	High	Low	High	C1
CPT	80	High	Abs	Medium	Abs	High	C1
CHA	580	Medium	High	Low	Abs	Low	C3
COL	3403	Low	Abs	High	Abs	Low	C2
DOU	580	Abs	High	Abs	Abs	Low	C3
ESG	580	Abs	High	Abs	Abs	Low	C3
ESS	580	Medium	High	Low	Abs	Low	C3
LEI	58	High	Abs	Medium	Abs	High	C1
MEB	580	Medium	High	Low	Abs	Low	C3
NUK	77	High	Abs	Low	Medium	Medium	C1
QUI	2850	High	Abs	Medium	Abs	High	C1
XHO	80	High	Abs	Medium	Abs	High	C1

To assess the significant effects of endogenous factors (age, sex, ear side) and exogenous factors (population, environment, language) on ear responses (including metrics such as mean amplitude, plateau amplitude, plateau start, plateau end, plateau median, and maximum frequency), we employed a linear mixed-effects model suitable for unbalanced designs^33^. A Box-Cox transformation was applied to frequency-related responses. We systematically tested variable interactions and statistically compared these models to their additive counterparts. Additionally, contrast analyses were conducted to examine subtle differences within variable sub-classes (e.g., sex: male and female, age: four categories)^34,35^. Post-hoc pairwise comparisons based on estimated marginal means (EMMs) identified statistically significant variables in the mixed-effects models. The statistical procedures utilised R packages: “nlme” for mixed-effects models, “emmeans” for contrast analysis, “MASS” for Box-Cox transformation, and “ggplot2” for graphical representations.

## Results

### Global analysis of TEOAE profiles in all populations

Anthropological data and TEOAE profiles were collected from 13 populations across five countries (Ecuador, England, Gabon, South Africa, and Uzbekistan), inhabiting diverse environments. The final dataset analysed comprised 448 individuals and 2,632 associated TEOAE profiles, evenly distributed between males and females within the 13 populations (Table 1). Averaged TEOAE profiles within the frequency range of 546.88 to 5117.19 Hz, corresponding to a median SNR > 3, were selected for further analysis (Fig. 1).

### Analyse of cochlear sensitivity variations between populations by class frequencies

The mean normalised amplitude curve was segmented into nine frequency classes for each population (Table 3 and Table 4) and the largest differences in amplitude between populations were calculated for each class. The most significant amplitude differences observed between the most disparate populations were: 4.75 dB for the [0.546–1.016[ class, 5.66 dB for the [1.016–1.523[ class, 8.72 dB for the [1.523–2.031[ class, 7.21 dB for the [2.031–2.5[ class, 5.84 dB for the [2.5–3.008[ class, 6.26 dB for the [3.008–3.516[ class, 4.49 dB for the [4.023–4.531[ class, and 4.28 dB for the [4.531–5.117[ class. These differences were observed between various pairs of populations (Table 3 and Table 4).

**Table 3:** Mean overall amplitude and sd (in normalized dB) for 9 selected frequency classes for each population studied (here from 546 to 2500 Hz).

		[ 0.546–1.016 [		[ 1.016–1.523 [		[ 1.523–2.031 [		[ 2.031–2.5 [	
		Mean	Sd	Mean	Sd	Mean	Sd	Mean	Sd
ECU	AND	-0,527	4,951	3,632	5,274	1,986	5,424	-0,653	5,233
ECU	COL	-0,961	4,698	2,180	6,006	-0,678	5,935	-2,924	5,647
ECU	QUI	-0,930	4,634	3,058	5,203	1,935	5,672	-0,406	5,434
ENG	LEI	-2,146	4,780	3,642	6,101	2,814	5,594	0,386	6,232
GAB	CHA	2,437	4,628	7,411	5,329	8,118	3,290	3,097	4,231
GAB	DOU	0,885	7,108	5,493	6,971	4,039	6,600	2,122	6,962
GAB	ESG	1,890	5,219	7,844	5,007	5,379	5,670	3,075	6,174
GAB	ESS	2,600	5,515	7,832	6,118	6,442	5,076	3,535	4,590
GAB	MEB	1,189	5,078	7,475	4,923	4,993	7,012	3,557	6,787
SA	CPT	1,623	5,229	7,527	5,510	6,427	5,917	4,291	5,778
SA	XHO	-0,794	5,619	4,268	7,231	5,096	6,897	3,231	6,416
UZB	BUK	-1,321	5,302	3,284	5,767	3,188	6,388	1,271	6,930
UZB	NUK	-0,994	5,234	4,436	5,222	2,844	5,185	0,531	5,171

**Table 4:** Mean overall amplitude and sd (in normalized dB) for 9 selected frequency classes for each population studied (here from 2500 to 5117 Hz).

		[ 2.5–3.008 [		[ 3.008–3.516 [		[ 3.516–4.023 [		[ 4.023–4.531 [		[ 4.531–5.117 [	
		Mean	Sd	Mean	Sd	Mean	Sd	Mean	Sd	Mean	Sd
ECU	AND	-2,275	5,647	-4,356	5,833	-6,063	5,853	-9,317	5,746	-13,253	5,463
ECU	COL	-3,461	5,283	-5,836	5,450	-8,597	5,451	-10,916	5,462	-14,696	4,986
ECU	QUI	-0,944	5,264	-3,053	5,559	-5,338	6,173	-7,304	6,468	-11,839	5,903
ENG	LEI	0,256	6,409	-0,271	5,715	-3,918	5,673	-7,913	5,728	-11,987	5,449
GAB	CHA	0,816	4,852	-2,158	4,408	-4,648	4,981	-7,329	4,942	-11,481	4,621
GAB	DOU	0,879	6,906	-0,613	6,722	-4,235	6,596	-8,114	5,807	-12,183	4,925
GAB	ESG	1,228	6,470	-1,943	6,742	-5,203	6,657	-8,190	5,271	-12,364	4,179
GAB	ESS	0,719	4,867	-2,555	4,935	-5,307	4,752	-9,712	4,521	-12,547	4,629
GAB	MEB	0,890	7,199	-1,696	6,261	-4,031	6,504	-6,431	7,543	-10,422	6,476
SA	CPT	2,376	5,101	0,418	5,575	-2,745	5,449	-6,377	6,101	-10,919	4,969
SA	XHO	1,000	7,782	-1,841	8,011	-5,311	7,457	-7,818	7,415	-11,858	5,722
UZB	BUK	-0,016	6,024	-1,501	6,280	-5,059	6,044	-8,405	5,870	-12,003	5,332
UZB	NUK	-0,148	5,296	-2,440	5,436	-5,351	5,580	-8,792	5,233	-13,187	4,372

### Analysis of acoustic metrics based on TEOAE profiles for all populations

We extracted and analysed from all TEOAE profiles the following acoustic metrics: the mean amplitude calculated across the entire profiles, the plateau characteristics (mean amplitude and start, median and end frequencies), and the maximal frequency, and those for each population. These metrics are schematically represented on Fig. 1 and values are provided in Table 5.

**Table 5:** Mean overall amplitude, mean plateau amplitude (in normalized dB), plateau characteristics (start, end, median in Hz) and frequency max (in Hz) for each population studied (on bold are mentioned the extreme values for each variable).

		Env class	Amplitude (Db)		Plat_amp (Db)		Plat start (Hz)		Plat end (Hz)		Plat med (Hz)		Freq max (Hz)	
			Mean	Sd	Mean	Sd	Mean	Sd	Mean	Sd	Mean	Sd	Mean	Sd
ECU	AND	C2	-3.72	3.91	6.7	5.6	1214.5	501.1	2087.1	811.6	1650.6	570.0	1699.5	715.9
ECU	COL	C2	**-5.40**	4.18	**4.8**	6.4	1110.6	423.5	1932.7	761.7	**1521.4**	492.0	1388.0	574.8
ECU	QUI	C1	-3.02	4.11	6.4	5.59	1316.20	589.4	2197.4	879.5	1756.6	618.8	1680.9	742.2
ENG	LEI	C1	-2.40	4.08	6.9	5.4	1379.4	547.6	**2372.1**	907.3	1875.5	618.9	1755.9	761.9
GAB	CHA	C3	-0.75	2.55	**11.1**	4.8	1193.1	322.5	1955.7	562.8	1574.2	357.7	1568.4	538.8
GAB	DOU	C3	-1.63	5.17	8.0	7.2	1272.2	535.0	2348.3	946.4	1810.0	651.6	**1865.8**	707.4
GAB	ESG	C3	-1.27	4.17	11.0	5.4	1210.2	390.3	**1898.1**	623.6	1553.9	443.6	1529.2	542
GAB	ESS	C3	-1.36	3.48	10.6	6.4	**1114.9**	274.5	2001.9	656.1	1558.2	348.0	**1349.3**	228.6
GAB	MEB	C3	**-0.80**	5.09	**11.1**	6.0	1310.2	373.3	2058.7	693.2	1684.2	429.8	1618.5	464.7
SA	CPT	C1	-0.04	3.59	10.7	5.4	1346.7	456.8	2224.7	844.5	1785.5	569.5	1623.9	568.7
SA	XHO	C1	-1.87	5.59	10.1	5.76	**1545.27**	590.0	2256.4	664.8	**1900.6**	543.3	1822.1	603.2
UZB	BUK	C1	-2.57	4.38	7.5	5.9	1404.5	568.9	2257.8	778	1830.94	595.69	1768.23	724.7
UZB	NUK	C1	-2.88	3.73	6.8	5.3	1242.6	458.0	2199.9	762.9	1721.0	495.0	1672.3	649.8

### Mean overall amplitude and plateau amplitude

The mean overall amplitude across TEOAE profiles ranged from -0.80 dB in Meubeum (MEU), Gabon, to -5.40 dB in Colta (COL), Ecuador. Similarly, the highest plateau amplitudes were observed in Meubeum (MEU; 11.1 dB) and Chateau (CHA; 11.12 dB) in Gabon, while the lowest plateau amplitudes were found in the Andes of Ecuador (4.84 dB in Colta, COL, up to 6.7 dB in Teligote, AND), suggesting potential influences of population or environmental variables on average amplitude variations among individuals. Both mean overall amplitude and plateau amplitude (spanning of maximal observed amplitude, in dB) provided similar insights, emphasising the significance of plateau features in the overall TEOAE response (Table 5).

### Frequency range of the plateau (start, end and median frequencies)

The acoustic characteristics of the plateau were analysed in detail: the plateau typically began around 1100 Hz (Gabon) and extended up to 1545 Hz (South Africa), marking variations of up to 500 Hz between populations. The plateau ended around 1898 Hz (Gabon) at the lower frequencies and up to 2372 Hz (England) at the higher frequencies. Median frequencies of the plateau ranged from 1521 Hz (Ecuador) to 1900 Hz (South Africa), reflecting significant variability among sampled individuals, as indicated by large standard deviations for these metrics.

### Frequency’s maximal amplitude

The frequency at which maximal amplitude was observed varied across populations, ranging from 1349 to 1865 Hz in Gabon.

### Assessing the relative impact of endogenous and exogenous factors

To identify factors influencing cochlear sensitivity in human populations, we employed mixed models to evaluate the relative impact of endogenous (biological factors such as sex, age, and ear side) versus exogenous factors. Initially, we considered population as an exogenous factor (a), and then categorised this into environmental classes based on objective ecological parameters defining each population’s landscape (3 classes; C1, C2, C3) (Tables 1 & 2). Additionally, we categorised individuals into four language families based on linguistic context rather than population units. Given their interrelated nature, we conducted separate mixed models for three independent models: (a) population-based, (b) environment-based, and (c) language-based models. We analysed seven parameters from TEOAE profiles: two amplitude metrics (whole amplitude and plateau amplitude, both in dB) and five frequency spectrum metrics (maximal frequency, plateau start, plateau median, plateau end, all in Hz). Of these, plateau width was found to be non-informative and excluded from further analyses.

Both amplitude metrics (whole and plateau) were influenced by both endogenous and exogenous factors across all models (Table 6). In all models (a, b, c), sex (endogenous) was the primary factor explaining inter-individual differences (F-value(a): 33.14, p-value < 0.0001; F-value(b): 33.65, p-value < 0.0001; F-value(c): 29.53, p-value < 0.0001). In the population-based model (a), age emerged as the second most influential factor affecting cochlear sensitivity (F-value: 11.9, p-value < 0.0001), followed by population (F-value: 6.5, p-value < 0.0001), and ear side (F-value: 5.20, p-value < 0.023). In both the environment-based model (b) and language-based model (c), the second most influential factor was the exogenous variable: environment (F-value: 22.82, p-value < 0.0001) and language (F-value: 14.59, p-value < 0.0001), respectively. However, using language as a grouping unit showed lower explanatory power than environmental factors in accounting for inter-individual variability. The ear side had the least impact (F-value: -0.39, p-value: 0.02) across all models. Plateau amplitude followed a similar pattern to the overall amplitude metrics (Table 6).

**Table 6:** Mixed models to define the relative impact of exogenous (population, environment or language) versus endogenous factors using TEOAE-data derived metrics (in bold are given significant values).

	Population model							
	Exogenous		Endogenous					
	Population		Age		Sex		Ear-side	
	F-value	p-value	F-value	p-value	F-value	p-value	F-value	p-value
Amp_avg_allspec	**6.46**	< .0001	**11.87**	< .000	**33.14**	< .0001	**5.20**	0.023
Ampl_plat	**7.72**	< .0001	**4.80**	0.003	**18.80**	< .0001	1.96	0.162
Freq_max	**2.00**	0.028	1.00	0.551	2.00	0.155	2.00	0.277
Fred_plat_ start	**3.00**	< .0001	0.00	0.744	1.00	0.291	2.00	0.178
Freq_plat end	**2.00**	0.025	1.00	0.451	0.00	0.749	1.00	0.458
Freq_plat med	**3.00**	0.001	1.00	0.523	0.00	0.982	1.00	0.241
Freq_plat width	0.95	0.500	1.13	0.335	0.38	0.536	0.18	0.671
	**Environment model (3 classes)**							
	Exogenous		Endogenous					
	**Environment**		**Age**		**Sex**		**Ear-side**	
	F-value	p-value	F-value	p-value	F-value	p-value	F-value	p-value
Amp_avg_allspec	**22.82**	< .0001	**11.01**	< .0001	**33.65**	< .0001	**5.27**	0.022
Ampl_plat	**22.38**	< .0001	**4.11**	0.007	**18.06**	< .0001	1.90	0.169
Freq_max	**4.00**	0.015	1.00	0.544	3.00	0.109	1.00	0.233
Fred_plat_ start	**10.00**	< .0001	1.00	0.447	2.00	0.195	2.00	0.152
Freq_plat end	**7.00**	0.001	1.00	0.243	0.00	0.818	0.00	0.723
Freq_plat med	**10.00**	< .0001	1.00	0.294	0.00	0.827	1.00	0.375
Freq_plat width	0.15	0.860	1.20	0.311	0.33	0.566	0.38	0.535
	**Language model (4 classes)**							
	Exogenous		Endogenous					
	**Language (4 classes)**		**Age**		**Sex**		**Ear-side**	
	F-value	p-value	F-value	p-value	F-value	p-value	F-value	p-value
Amp_avg_allspec	**14.59**	< .0001	**11.38**	< .0001	**29.53**	< .0001	**5.02**	0.026
Ampl_plat	**19.47**	< .0001	**4.76**	0.0028	**15.55**	0.0001	1.73	0.189
Freq_max	**3.00**	0.0156	1.00	0.374	2.00	0.157	1.00	0.23
Fred_plat_ start	**3.00**	0.0438	1.00	0.3162	1.00	0.277	2.00	0.136
Freq_plat end	**3.00**	0.0312	2.00	0.1629	0.00	0.710	0.00	0.749
Freq_plat med	**4.00**	0.0135	2.00	0.1799	0.00	0.988	1.00	0.358
Freq_plat width	0.66	0.5763	1.25	0.292	0.34	0.557	0.39	0.534

Frequency metrics (Frequency_max, Freq_plateau start, Freq_plateau median, and Freq_plateau end) were exclusively influenced by exogenous factors; all endogenous factors were non-significant across all individual grouping levels (population, environment, or language). Overall, these findings underscore the significant impact of biological factors on amplitude metrics, complemented by exogenous factors, whereas frequency metrics appear exclusively influenced by exogenous factors.

### Contrast analyses and nuanced differences between sub-classes

To delve deeper, we employed contrast analyses to examine nuanced differences between sub-classes of each analysed variable (Table 7).

**Table 7:** Contrast analyses : amplitude parameter in a population-based model (**a**); amplitude and frequency metrics parameters in an environment-based model **(b)** with C1: urban samples, C2: rural high-altitude samples C3: rural protected forest samples, ; and amplitude parameter in a language-based model **(c).** For population analysis, only the three first significant population pairs are given; Full data contrasts are available in supplementary material 3 (supp. table 3).

	Amplitude in population-based model (a)					Amplitude in language-based model (c)				
	Sub-classes	Estimate	SE	t-ratio	*p-value*	Sub-classes	Estimate	SE	t-ratio	*p-value*
Pop/Lang	COL-CPT	-4.95	0.75	-6.60	**9.3E-09**	Amer-NigCong	**-4.10**	0.68	-6.06	** < 0.0001**
	COL-MEB	**-5.11**	0.98	-5.19	**2.4E-05**	Amer-IndEu	-2.55	0.65	-3.90	**0.0006**
	CHA-COL	4.37	0.91	4.82	**1.4E-04**	IndEu-NigCong	-1.54	0.41	-3.74	**0.0012**
Sex	F-M	1.96	0.34	5.76	**1.60E-08**	F-M	1.90	0.35	5.43	** < 0.0001**
Age	18–25 vs 25–35	0.85	0.54	1.57	0.39	18–25 vs 25–35	0.45	0.51	0.88	0.813
	18–25 vs 35–45	2.26	0.54	4.20	**0.0002**	18–25 vs 35–45	2.14	0.53	4.06	**0.0003**
	18–25 vs 45–55	2.96	0.62	4.77	** < 0.0001**	18–25 vs 45–55	2.59	0.60	4.32	**0.0001**
	25–35 vs 35–45	1.41	0.43	3.25	**0.0067**	25–35 vs 35–45	1.69	0.43	3.89	**0.0007**
	25–35 vs 45–55	2.14	0.53	3.98	**0.0005**	25–35 vs 45–55	2.14	0.53	4.06	**0.0003**
	35–45 vs 45–55	0.70	0.54	1.29	0.560	35–45 vs 45–55	0.45	0.55	0.84	0.8442
Ear-side	L-R	-0.38	0.17	-2.21	**0.027**	L-R	**-0.38**	0.17	-2.24	0.03
	**Environment-based model (b) : whole amplitude**					**Environment based-model (b) : frequency spectrum**				
	**Sub-classes**	**Estimate**	**SE**	**t-ratio**	***p-value***	**Sub-classes**	**Estimate**	**SE**	**t-ratio**	***p-value***
Env	C2-C3	**-3.57**	0.54	-6.64	**2.54E-10**	C1—C2	0,005	0,001	4,26	**0,0001**
	C1-C2	2.31	0.47	4.97	**2.81E-06**	C1—C3	0,003	0,001	2,43	**0,0411**
	C1-C3	-1.25	0.42	-3.00	**7.99E-03**	C2—C3	-0,003	0,001	-1,81	0,1665
Sex	F-M	2.01	0.35	5.80	**1.24E-08**	**Plateau med**				
Age	18–25 vs 25–35	0.42	0.49	0.86	0.827	C1—C2	0,008	0,002	4,28	**0,0001**
	18–25 vs 35–45	2.03	0.52	3.90	**0.001**	C1—C3	0,004	0,002	2,61	**0,0251**
	18–25 vs 45–55	2.56	0.59	4.34	**0.000**	C2—C3	-0,004	0,002	-1,69	0,2115
	25–35 vs 35–45	1.61	0.43	3.73	**0.001**	**Plateau end**				
	25–35 vs 45–55	2.14	0.52	4.12	**0.000**	C1—C2	0,012	0,004	3,48	**0,0016**
	35–45 vs 45–55	0.53	0.54	0.98	0.760	C1—C3	0,007	0,003	2,25	0,0643
Ear-side	L-R	**-0.39**	0.17	-2.29	0.022	C2—C3	-0,005	0,004	-1,27	0,4116

**Fig. 2:**
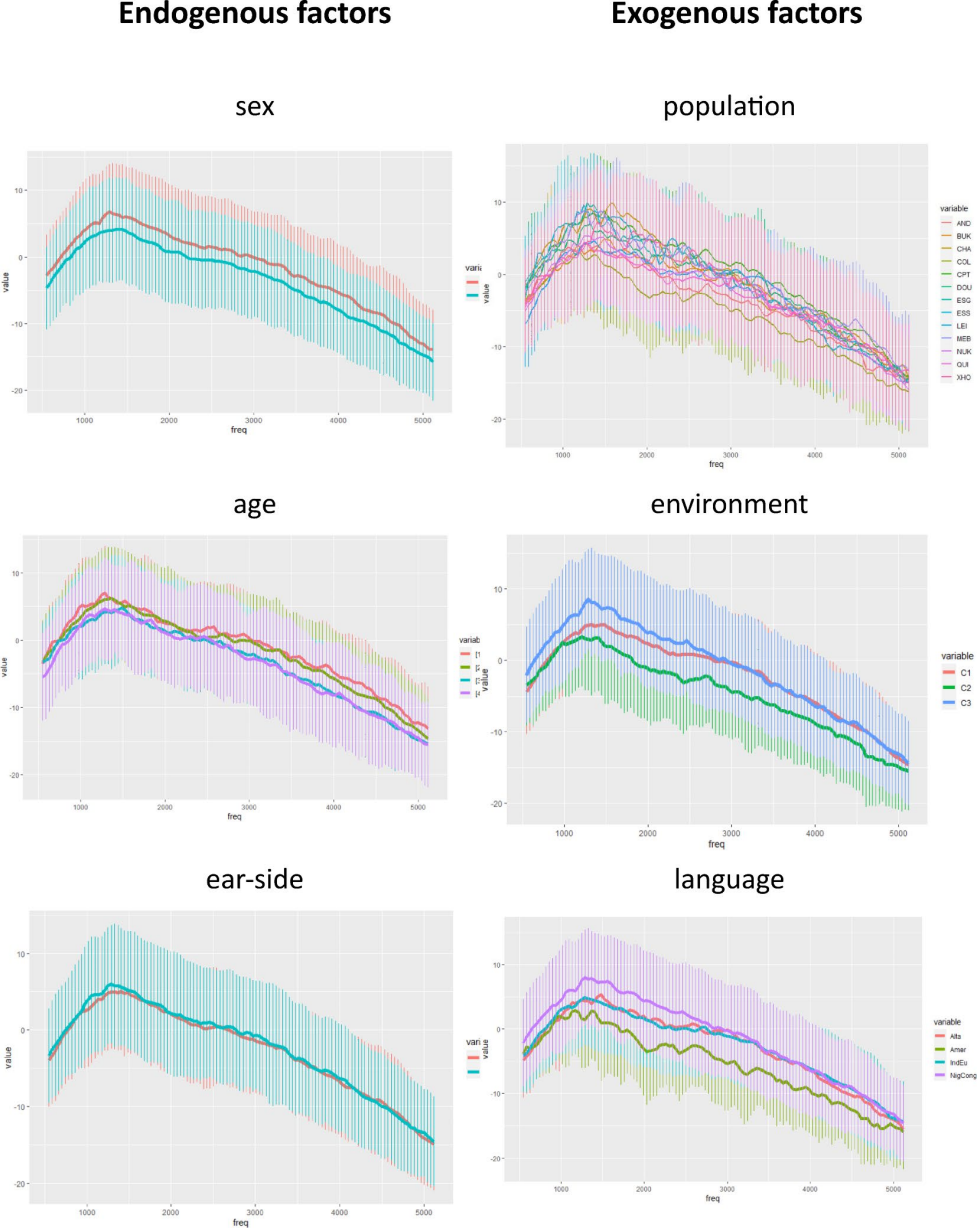
Mean TEOAE profile represented using all factors tested: for endogenous factors sex, age, ear-side, and for exogenous factors, populations, environments and languages.

In the population-based model (a), for amplitude metrics, strong contrasts were observed between population pairs, particularly those including Amerindian populations (Table 7 and supplementary material 3, supp. table 3). Sex also contributed significantly, explaining around 2 dB differences between men and women. Age sub-classes showed estimates ranging from 1.4 to 3 dB, with notable homogeneity observed between the youngest (18–25 and 25–35 years) and oldest (35–45 and 45–55 years) age groups, suggesting significant age-related trends (Fig. 2). Ear side contributed subtly to this variation (estimate: -0.4 dB).

In the environment-based model (b), populations were reclassified into three environmental sub-classes based on key ecological parameters (altitude, forest coverage, vegetation diversity, level of artificialisation, bare soil coverage, and population density). This allowed us to separate populations speaking different languages but residing in similar environmental contexts, thereby mitigating the confounding effects of language.

We found that environment played a pivotal role in shaping the amplitude of TEOAE profiles (estimates ranging from -3.6 to 1.2 dB), followed by sex (estimate: 2 dB) and age sub-classes (estimates ranging from 2.5 to 0.4 dB), with ear laterality having the least impact (estimate: -0.4 dB). Notably, significant differences were observed between C2 (rural high-altitude) and C3 (rural protected forest), with C2 exhibiting the highest and C3 the lowest amplitudes (C2 and C3: -3.6 dB, p-value < 0.0001).

Environment played a crucial role in modelling the frequency spectrum within the plateau: C1 (urban) and C2 (rural high-altitude) showed significant contrasts across the entire plateau (F-value: 0.005, p-value: 0.0001; F-value: 0.012, p-value: 0.0016; F-value: 0.008, p-value: 0.0001), while C1 (urban) and C3 (rural natural forest) exhibited significant contrasts in plateau start and median (F-value: 0.003, p-value: 0.04; F-value: 0.004, p-value: 0.025), with no significant differences observed between C2 and C3. Consequently, the most discriminative environment was C1 (urban) compared to C2 (rural high-altitude) and C3 (rural protected forest), suggesting distinct frequency spectrum observed in urban populations compared to those residing in protected areas or extreme altitudinal zones.

Exploring the potential impact of language, we applied a language-based model (c), substituting populations with their respective language families: Amerindian, Niger-Congo, Indo-European, and Turko-Mongol (Table 6). The most contrasted pair of language families was observed between Amerindian and Niger-Congo (F-value: -4.10, p-value: < 0.0001). A similar contrast pattern was observed as in the previous models for endogenous factors such as sex, age, and ear side (Table 6).

## Discussion

**Fig. 3:**
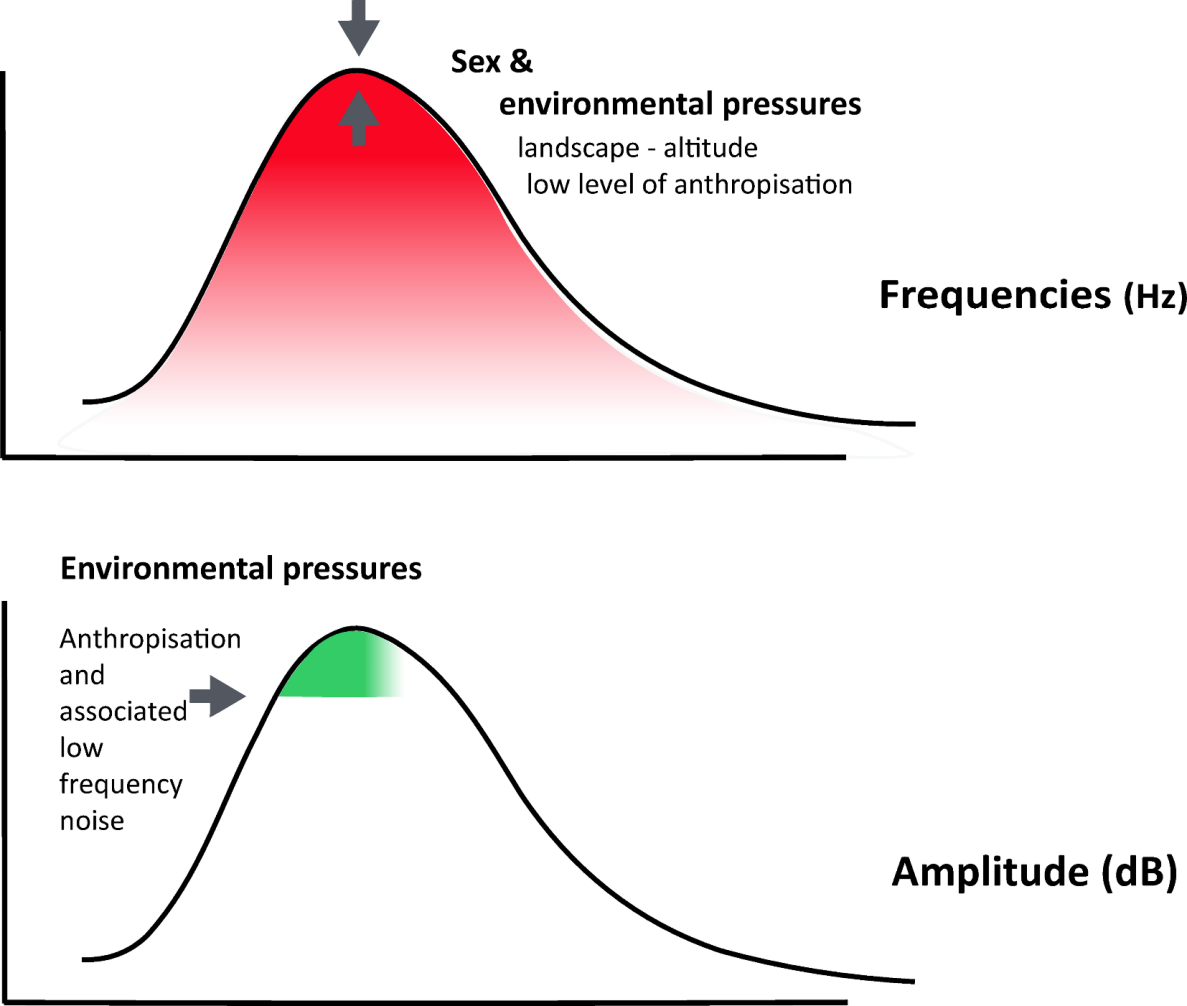
Endogenous and exogenous factors modeling hearing sensibility.

Our study provides new insights into the factors that influence cochlear sensitivity across human populations, measured by transient-evoked oto-acoustic emissions. By challenging established concepts primarily derived from european studies, we offer fresh perspectives on the role of ecological environments in shaping the human cochlear sensitivity. Our findings show that amplitude metrics are influenced by both endogenous factors (such as sex, age, and ear side) and exogenous factors, whereas frequency spectrum metrics are shaped exclusively by exogenous factors. These results suggest that environmental influences play a crucial role in determining the range of frequencies individuals’ ears can “perceive” within different environments (Fig. 3).

**Sex** emerges as the most influential factor shaping variation in TEOAE amplitude among the endogenous factors examined (age, sex, ear side). While sex-specific differences in cochlear sensitivity have been suggested in previous studies, they have typically been observed at frequencies around and above 2 kHz ^24–26,36^. Our study, however, reveals that women consistently exhibit higher sensitivity by 2 dB in average across the entire frequency spectrum tested in all populations sampled (and up to 6 kHz in some populations).

Several hypotheses can help explain these findings. First, sex differences in OAEs have proposed to be partly attributable to varying levels of androgen exposure during prenatal development^25,37,38^. Second, structural differences in cochlear anatomy between men and women have been observed in specific cochlear segments in both European and African populations^39^; although these do not fully account for the observed differences across the entire frequency range analysed, suggesting either that CT scans failed to detect fine sex-specific morphological variations in the whole cochlear structure or the existence of other factors. Alternatively, sex-specific differences in outer hair cell function, including organization, density, or sensitivity, could underpin these variations, potentially influenced by sex-specific regulatory mechanisms, as seen in other organs^40^.

In addition to heightened cochlear sensitivity, women also perform better in other acoustic tests, such as PTA, SOAEs, CEOAEs, ABR, and speech perception, indicating superior function in both the peripheral auditory system and the central auditory pathways^36^. Furthermore, women show a higher prevalence of hyperacusis^41^, suggesting a heightened responsiveness to sensory stimuli without significant down-regulation. Acoustic overstimulation activates complex cellular responses within the cochlea, triggering the antigen-presenting function of cochlear mononuclear phagocytes, which could link innate and adaptive immune responses^42^. Given the well-documented detrimental effects of noise on overall health, such as reduced sleep quality and increased cardiovascular disease risk^43^, maintaining heightened cochlear sensitivity in noisy environments may entail evolutionary trade-offs. The observed trend for women to perceive auditory signals more accurately and to be more attuned to sensory environments^44,45^ may reflect broader trade-offs within sensory systems or at the whole-body level. These sex differences are also relevant in the context of biological ageing, as, despite women’s longer lifespans, they often experience poorer health outcomes in later life^46^, raising questions about their overall quality of life. While the precise factors explaining why women tend to have higher cochlear sensitivity remain debated, our results provide compelling evidence of this hypersensitivity across the entire frequency spectrum analysed (500–5500 Hz) and across all populations sampled. This offers strong support for a consistent and robust sex-based difference in cochlear sensitivity, with potential implications for understanding sensory processing and health in diverse environments. 

**Age** is a well-established factor in the decline of cochlear sensitivity. However, in our study, the effect of age is overshadowed by sex and environmental factors. The amplitude of TEOAE curves gradually decreases between the ages of 18 and 55. Contrast analyses reveal a significant decline between the youngest age groups (18–25, 25–35) and older groups (35–45, 45–55), indicating a more rapid hearing loss starting around 35 years of age in healthy populations. This suggests an earlier onset of cochlear sensitivity decline compared to other studies^47^ but is consistent with the age threshold for hearing decline observed in carriers of the p.Pro51Ser variant^48^.

**Ear-side** is the least influential endogenous factor in explaining hearing variability among individuals in our analyses. While extensively studied to determine ear dominance^49^, typically favouring the right ear, our study shows that ear-side has only a marginal effect on cochlear sensitivity. Nevertheless, our results reaffirm a consistent, albeit subtle, right ear advantage across populations. This right ear advantage, excluding the contralateral effect, has been consistently observed in several previous studies^22,50,51^. This is likely due to the right ear’s primary role in auditory processing within the left hemisphere, which is crucial for human abilities such as speech perception and production^22^. This advantage for speech perception with tonal stimuli^52–54^ and preference for noisy environments^23^ seems to be a universal sensory trait across human populations, regardless of ethnicity, ecological context, or language. Furthermore, our findings indicate a more pronounced decline in cochlear sensitivity in the left ear compared to the right, particularly after the age of 45. This could suggest a quicker relaxation of functional constraints in the left ear over the lifespan. Combined with evidence of stronger heritability in the right ear^55^, these results imply i) that functional pressures (and at least partially, evolutionary pressures) may play a larger role in preserving better acuity in the right ear and ii) that left ear sensitivity would be more driven by environmental pressures.

Our results offer pioneering insights into the role of **exogenous** (non-biological) factors in shaping cochlear sensitivity. By comparing 13 contrasted populations, we have demonstrated that exogenous factors—classified as population, environment, or language—significantly contribute to variations in cochlear sensitivity across human groups. The environment, in particular, not only influences the amplitude of the cochlear response, as endogenous factors do, but also affects the range of frequencies that can be “perceived”.

Several hypotheses could explain the differences in mean hearing profiles observed across the three environmental categories: (i) physiological adaptations affecting the entire body system^56–58^ —not just the auditory system—in response to factors such as elevation^59^ and temperature^18^, which may indirectly influence cochlear sensitivity; (ii) long-term acoustic adaptations to varying soundscapes, characterised by intrinsic differences in noise intensity, sound types, sound propagation, and attenuation^60–64^ (see AAH theory and derived theories); and (iii) the impact of varying levels of anthropogenic activity^65^, including exposure to chemical compounds that damage hair cells^1,66^. Since both noise exposure and many chemical agents share similar ototoxic mechanisms, combined exposure to these factors may exacerbate hearing loss^67^.

We analysed amplitude metrics through contrast analyses, revealing significant differences across all environmental classes. The greatest contrast was found between populations living in protected forest environments (C3) and high-altitude Andean populations (C2) in Ecuador, with a 5–7 dB deviation. The C3 class, characterised by tropical environments with rich biophony and minimal anthropophony, suggests that higher cochlear sensitivity in these populations may reflect either an innate sensitivity to minimally anthropized environments or an inherited trait favouring heightened sensitivity in biophonic environments, where vigilance is essential for survival^68,69^.

In the C2 class, the lowest TEOAE amplitudes were observed in rural populations living at high altitudes (Teligote, AND, and Colta, COL). This reduced sensitivity could be due to: (i) the effect of atmospheric pressure on TEOAE measures; (ii) potential sound attenuation in high-altitude environments, which might lower the need for high auditory acuity; or (iii) physiological adaptation to hypoxia. However, as no eco-acoustic studies at high altitudes have reported sound attenuation, this hypothesis remains speculative and warrants further investigation.

 Previous studies reported a reduction of auditory function with high-altitude (for a review see Masè^70^). Regarding the impact of atmospheric pressure on TEOAE measures, previous studies reported a decrease of 0.23 dB/kPa in the lower frequency range (500 Hz to 2 kHz), with negligible attenuation above 2 kHz^71^. However, our results challenge these findings. The 2,500-m altitudinal difference between the most extreme populations in our study, corresponding to a 25 kPa pressure difference, is inconsistent with the observed results. Furthermore, the attenuation of the entire TEOAE profile suggests the involvement of additional factors, such as the middle-ear specific role, among others.

High-altitude hypoxic stress is known to induce physiological changes^72^, such as increased blood flow and brain circulation^73^, particularly in populations like Tibetans^74,75^. Neurobehavioral alterations affecting memory and learning have also been reported^76^. Given that more than 13% of youth in high-altitude areas experience hearing loss^77^ and studies in rats show cochlear hair cell damage due to hypoxia^78^, long-term adaptation to low oxygen may contribute to the observed lower cochlear sensitivity. Damage to outer hair cells, which play a key role in TEOAE, occurs first at the cochlear base and much less at the apex^78^. This is consistent with our data, where low-frequency TEOAE responses are preserved compared to medium and high frequencies in rural high-altitude (C2) populations versus urban (C1) populations. Further research on TEOAE in diverse high-altitude populations, considering different ethnic backgrounds, would provide more insights. Finally, testing the effect of language revealed similar patterns to those seen with environmental factors, aligning with recent studies linking temperature and language sonority^18^.

We analysed frequency spectrum metrics using contrast analyses and found that urban populations (C1) differ significantly from both protected forest (C3) and high-altitude (C2) populations, particularly in the initial and middle plateau metrics, while C2 and C3 populations show no significant differences. This suggests that environmental factors shape urban populations (C1) distinctively from rural populations (C2 and C3). The shift towards higher frequencies in the TEOAE plateau observed in C1 populations might be a response to the low-frequency traffic noise prevalent in urban environments, similar to the shifts seen in bird vocalisations in anthropized areas^79–71^.This results establishes a basis for further research into how urban environments affect the senses of living organisms, including but not limited to humans.

This study highlights the crucial influence of environmental factors on cochlear sensitivity and underscores the need for further research to determine, more specifically, whether phenotypic plasticity alone or also evolutionary genetic adaptations are involved. For instance, urban environments may be too recent for genetic adaptation to explain differences in cochlear sensitivity compared to rural areas, making adaptive or non-adaptive phenotypic plasticity the more plausible explanation. In contrast, altitude-related differences may follow a different pattern, as populations living at high altitudes have developed physiological adaptations to low-oxygen environments through ancient genetic adaptation^74^. These adaptations could involve trade-offs with other physiological functions and traits, including auditory sensitivity and would benefit from furthers investigations.

## Conclusion

By examining both endogenous and exogenous factors, our study sheds light on the key parameters shaping cochlear sensitivity across human populations. We demonstrate that understanding global variations in cochlear sensitivity requires considering not only traditional biological factors but also the ethnic and ecological diversity of populations. Our findings highlight the importance of integrating both ‘population’ and ‘environment’ to uncover subtle variations in cochlear sensitivity. These factors influence not only TEOAE amplitude but also the frequency range perceived in different environments, a dimension often overlooked in hearing research. Identifying the drivers behind natural variation in cochlear sensitivity will improve our understanding of hearing loss, hyperacusis, and individual differences in noise tolerance. This comprehensive approach is essential for developing targeted prevention strategies and tailoring hearing aids to diverse populations.

## Supplementary Information

Supplementary Information 1.

Supplementary Information 2.

Supplementary Information 3.

## Data Availability

TEOAE-profiles were securely stored in a private dedicated PostgreSQL database using anonymous individual codes in agreement with the French ethical committee and RGPD guidelines. TEOAE derived-metrics were generated as explained and are accessible in supplementary material 2 (supp. table 2). TEOAE derived-metrics are provided in supplementary material (supp. table 3).
