# Supplementary material for: Sex and environment shape cochlear sensitivity in human populations worldwide
Source: Sci Rep. 2025 Mar 26;15:10475. doi: 10.1038/s41598-025-92763-6 (PMC11947323; doi:10.1038/s41598-025-92763-6)
Supplement: Supplementary file 1 — Supplementary Information 1. [file 41598_2025_92763_MOESM1_ESM.docx]

*Supplementary material for*

**Sex and environment shape cochlear sensitivity in human populations worldwide**

Patricia Balaresque Ph.D*^1^, Sébastien Delmotte Ph.D^2^, Franklin Delehelle Ph.D^3,11^, Andreia Moreira Ph.D^3^, Nancy Saenz-Oyhéréguy Ph.D^4^, Myriam Croze Ph.D^5,12^, Tatyana Hegay Ph.D^6^, Tamara Aripova Ph.D^6^, Sylvie Le Bomin Ph.D^7,13^, Philippe Mennecier Ph.D^7^, Didier Descouens M.D^8^, Sylvain Cussat-Blanc Ph.D^3,14^, Hervé Luga Ph.D^3,15^, Angel Guevara Ph.D^16^, Maria Eugenia D’Amato Ph.D^5^, Turi King Ph.D^9^, Catherine Mollereau Ph.D^10^ and Evelyne Heyer Ph.D^7^

Corresponding author: Patricia Balaresque*^1^

**MATERIAL AND METHODS**

Here is the schematic representation of the experimental apparatus Echoport ILO288 USB-I Otodynamics engine (Amplifon) used to collect TEOAE. A unique probe (Unique ID: 0000014B5513) was consistently used for all measurements (**Supplementary Figure 1**).


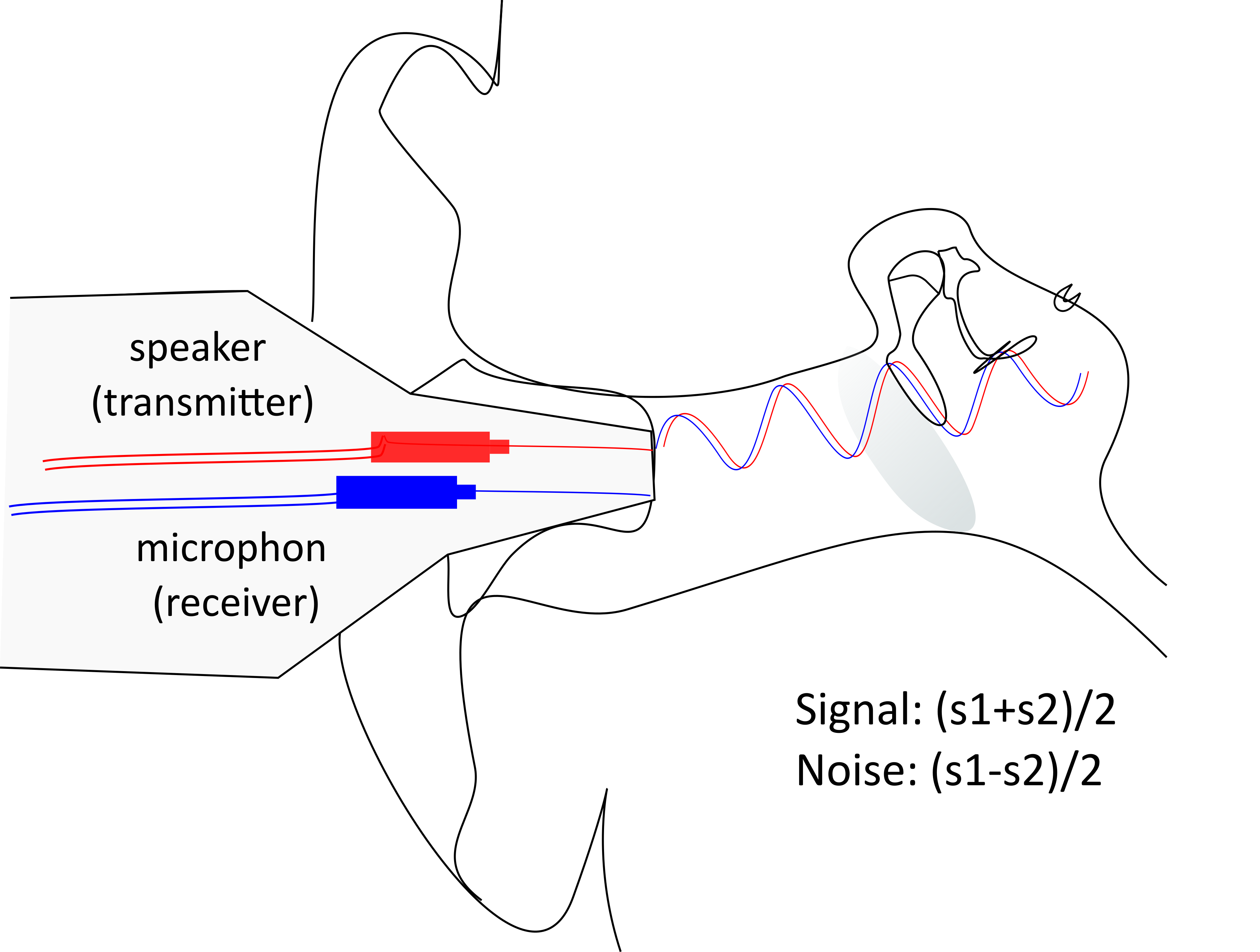


**Supplementary Figure 1**: Schematic TEOAE experimental apparatus

**Pre-processing of the data:** Each TEOAE measurement generates a DTA file exportable from the Echoport ILO288 USB-I otodynamics machine and readable using a script provided by the manufacturer (originally by Amplifon, subsequently improved for easier raw data visualization). The raw dataset initially comprised 2688 TEOAE measurements corresponding to 450 individuals across 13 sampled populations. Before conducting statistical analyses, the raw data underwent preprocessing. The first step involved identifying atypical audiograms likely affected by external noise or physiological issues that went undetected. For each individual and each ear, cross-correlation (or wave reproducibility) was calculated among triplicate TEOAE measurements with a lag of 0 to ensure similarity of all collected signals per ear. TEOAE measurements with cross-correlation values below 0.7 compared to others were visually inspected. Audiograms that significantly deviated in shape and potentially affected the mean TEOAE signal were excluded, and cross-correlation was recalculated. In total, eight TEOAE measurements were excluded. One individual from the COL group, showing markedly different audiograms suggesting possible external noise or undetected physiological issues, was completely excluded from the analyses. This process resulted in a final dataset of 448 individuals with 2632 audiograms: 49 individuals (25 females and 24 males distributed across the 13 populations) had measurements from only one ear (20 left and 29 right). Although this led to an unbalanced design, excluding these individuals did not significantly alter the results. Therefore, individuals with measurements from only one side of TEOAEs were retained to avoid biasing certain populations (primarily AND and CPT, with 7 and 8 individuals respectively).

|  | **AND** | **BUK** | **CAP** | **CHA** | **COL** | **DOU** | **ESG** | **ESS** | **LEI** | **MEB** | **NUK** | **QUI** | **UWC** | **XHO** |
| --- | --- | --- | --- | --- | --- | --- | --- | --- | --- | --- | --- | --- | --- | --- |
| F | 17 | 21 | 9 | 11 | 22 | 16 | 12 | 7 | 20 | 12 | 27 | 23 | 10 | 11 |
| M | 12 | 23 | 7 | 11 | 19 | 8 | 13 | 4 | 18 | 6 | 21 | 22 | 11 | 6 |

|  | **[18-25[** | **[25-35[** | **[35-45[** | **[45-55]** |
| --- | --- | --- | --- | --- |
| F | 43 | 92 | 70 | 37 |
| M | 38 | 76 | 56 | 36 |

**Supplementary Table 1**: **Detailed sample sizes (sex and age)**

**Questionnaires and data storage:**

At the conclusion of the data collection campaign, questionnaires containing biological, environmental, and cultural information were grouped with the aforementioned TEOAE records of each subject, then entered into a final dedicated PostgreSQL database that consolidated the entire dataset. This database allows for flexible querying, filtering, and extraction of data for further analysis. Collected data were securely stored in a local database using anonymous individual codes. Anonymized data will be made available on Dryad.

**SUPPLEMENTARY MATERIAL legends**

**Supplementary material 1 – supp. figure 1**: Schematic TEOAE experimental apparatus

**Supplementary material 1 – supp. table 1:** Detailed effective sizes (sex and age)

**Supplementary material 2 – supp. table 2:** All individuals TEOAE-derived metrics

**Supplementary material 3 – supp. table 3:** Detailed contrast analyses
